# Molecular characterization of the missing electron pathways for butanol synthesis in *Clostridium acetobutylicum*

**DOI:** 10.1038/s41467-022-32269-1

**Published:** 2022-08-10

**Authors:** Céline Foulquier, Antoine Rivière, Mathieu Heulot, Suzanna Dos Reis, Caroline Perdu, Laurence Girbal, Mailys Pinault, Simon Dusséaux, Minyeong Yoo, Philippe Soucaille, Isabelle Meynial-Salles

**Affiliations:** 1 TBI, Université de Toulouse, CNRS, INRAE, INSA, Toulouse, France; 2 BBSRC/EPSRC Synthetic Biology Research Centre, School of Life Sciences, Centre for Biomolecular Sciences, University of Nottingham, Nottingham, UK

**Keywords:** Metabolic engineering, Applied microbiology, Biocatalysis

## Abstract

*Clostridium acetobutylicum* is a promising biocatalyst for the renewable production of *n*-butanol. Several metabolic strategies have already been developed to increase butanol yields, most often based on carbon pathway redirection. However, it has previously demonstrated that the activities of both ferredoxin-NADP^+^ reductase and ferredoxin-NAD^+^ reductase, whose encoding genes remain unknown, are necessary to produce the NADPH and the extra NADH needed for butanol synthesis under solventogenic conditions. Here, we purify, identify and partially characterize the proteins responsible for both activities and demonstrate the involvement of the identified enzymes in butanol synthesis through a reverse genetic approach. We further demonstrate the yield of butanol formation is limited by the level of expression of *CA_C0764*, the ferredoxin-NADP^+^ reductase encoding gene and the *bcd* operon, encoding a ferredoxin-NAD^+^ reductase. The integration of these enzymes into metabolic engineering strategies introduces opportunities for developing a homobutanologenic *C. acetobutylicum* strain.

## Abstract (web summary)

Ferredoxin-NAD(P) + oxidoreductases are important enzymes for redox balancing in *n*-butanol production by *Clostridium acetobutylicum*, but the encoding genes remain unknown. Here, the authors identify the long sought-after genes and increase *n*-butanol production by optimizing the levels of the two enzymes.

## Introduction

C*lostridium acetobutylicum* is a Gram-positive, spore-forming anaerobic bacterium capable of converting various sugars and polysaccharides to organic acids (acetate and butyrate) and solvents (acetone, butanol, and ethanol). Due to its importance in the industrial production of the bulk chemicals acetone and butanol^1–3^ and its potential use in the production of *n*-butanol, a promising biobased liquid fuel with several advantages over ethanol^4,5^, much research has focused on (i) understanding the regulation of solvent formation^6–15^ and (ii) metabolically engineering this microorganism to produce high yields of alcohols^16–18^.

**Fig. 1:**
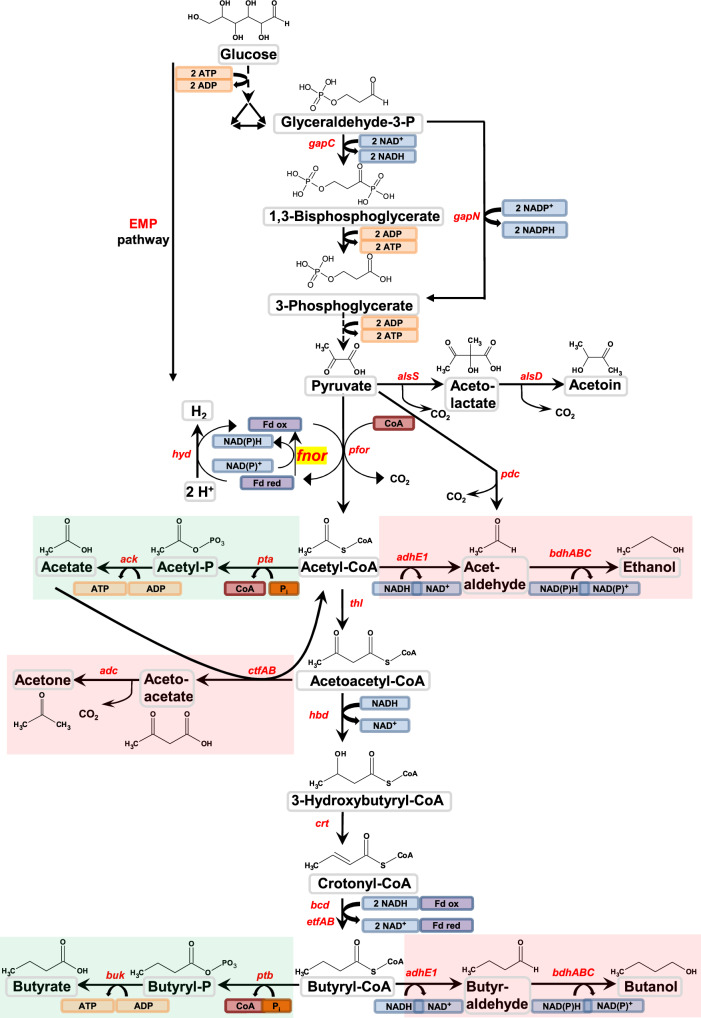
Central metabolism of *Clostridium acetobutylicum*. The green box indicates the primary products under acidogenic conditions, whereas the red box indicates the primary products under solventogenic conditions. The letters in red and italics indicate the corresponding genes. *ack* acetate kinase, *adc* acetoacetate decarboxylase, *adhE1* aldehyde dehydrogenase, *adhE2* bifunctional aldehyde/alcohol dehydrogenase, *alsD* alpha-acetolactate decarboxylase, *alsS* acetolactate synthase, *bcd* butyryl-CoA dehydrogenase, *bdh* butanol dehydrogenase, *buk* butyrate kinase, *crt* crotonase, *ctfAB* CoA-transferase, *etf* electron transfer flavoprotein, *hbd* 3-hydroxybutyryl-CoA dehydrogenase, *hyd* hydrogenase, *fnor* ferredoxin-NAD(P)^+^ oxidoreductases, *pdc* pyruvate decarboxylase, *pfor* pyruvate:ferredoxin oxidoreductase, *pta* phosphotransacetylase, *ptb* phosphotransbutyrylase, *thl* thiolase, *gapC* NADH-dependent glyceraldehyde-3-phosphate dehydrogenase, *gapN* nonphosphorylating NADPH-producing glyceraldehyde-3-phosphate dehydrogenase, Fd ox represents oxidized ferredoxin, whereas Fd red represents reduced ferredoxin.

**Fig. 2:**
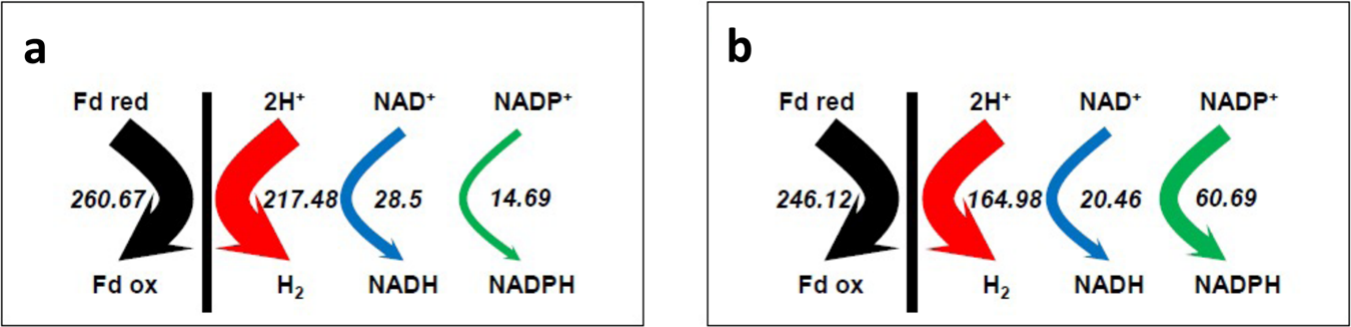
Electron flux analysis of *C. acetobutylicum* in phosphate-limited chemostat cultures. Under acidogenic (pH 6.3) (**a**) and solventogenic (pH 4.4) (**b**) conditions. All values (mmol/gDCW/h) are normalized to the flux of glucose consumption. Data were extracted from ref. 19. The black arrow represents the total flux of Fd_red_ oxidation, the red arrow the hydrogenase flux, the blue arrow the Ferredoxin-NAD^+^ flux, and the green arrow the ferredoxin-NADP^+^ flux.

Using a global system biology approach to the characterization of the solventogenic metabolism of a phosphate-limited chemostat culture of *C. acetobutylicum*, the six steps involved in the conversion of acetyl-CoA to butanol (Fig. 1) were previously characterized^19^: the main enzyme responsible for crotonyl-CoA reduction to butyryl-CoA was shown to be the BCD complex (encoded by *bcd*, *etfB*, and *etfA*, the *bcd* operon), a bifurcating enzyme consuming two moles of NADH and producing one mole of reduced ferredoxin, and the last two steps of butanol production were shown to be catalyzed by AdhE1 through its NADH-dependent aldehyde dehydrogenase activity and by BdhB, BdhC, and BdhA through their NADPH-dependent butanol dehydrogenase activity. These results had a strong impact on electron flux distribution, as it was demonstrated that both ferredoxin-NADP^+^ reductase and ferredoxin-NAD^+^ reductase activities were necessary to produce the NADPH and the extra NADH needed for butanol synthesis from acetyl-CoA (Fig. 2)^19^. Although the activities of these enzymes were previously detected in *C. acetobutylicum*^6,20^, the encoding genes remained unknown^19,21^. Ferredoxin-NADP^+^ reductase enzymes (FNOR) (EC 1.18.1.3) are distributed over a variety of aerobic organisms from prokaryotes to eukaryotes, especially in plants^22^, but have never been purified and characterized from any clostridial species. In contrast, ferredoxin-dependent NAD^+^ reduction coupled to proton export (Rnf) and ferredoxin-dependent transhydrogenases (Nfn) have been characterized from a molecular perspective in several clostridial species but no homologs have been found in *C. acetobutylicum*^23–25^. Moreover, *C. acetobutylicum* was shown to be unable to produce NADPH by the oxidative pentose-phosphate pathway, as a gene encoding a glucose-6-P dehydrogenase, the first and key enzyme of this pathway, was missing^19,26^.

In this work, we purify and identify the proteins responsible for the ferredoxin-NAD^+^ and ferredoxin-NADP^+^ reductase activities in *C. acetobutylicum* under solventogenic conditions. We further confirm their essential physiological role in butanol synthesis in *C. acetobutylicum* and demonstrate that butanol production is limited by the electron flux between reduced ferredoxin and both NADP^+^ and NAD^+^. By just overexpressing the ferredoxin-NADP + encoding gene, it is possible to produce butanol at yield that have only been obtained before by an extensive metabolic engineering approach^18^.

## Results

### Biochemical analysis of the glucose to *n*-butanol pathway in *C. acetobutylicum*

*C. acetobutylicum* can, in theory, convert one mole of glucose into one mole of *n*-butanol. The conversion of one mole of glucose to two moles of acetyl-CoA is associated with the production of two moles of NADH in the EMP (Embden–Meyerhof–Parnas) pathway and two moles of reduced ferredoxin during the decarboxylation of two moles of pyruvate to two acetyl-CoA using pyruvate ferredoxin oxidoreductase (PFOR) (Fig. 1). However, under solventogenic conditions, the conversion of two moles of acetyl-CoA to one mole of *n*-butanol produces one mole of reduced ferredoxin during the reduction of crotonyl-CoA to butyryl-CoA by butyryl-CoA dehydrogenase (BCD) and consumes four moles of NADH and one mole of NADPH to reduce butyraldehyde to *n*-butanol (Fig. 1) by the NADPH-dependent alcohol dehydrogenases BdhB, BdhC and BdhA^19^.

As the EMP pathway produces less NADH than the *n*-butanol pathway consumes, for each mole of *n*-butanol produced, two moles of reduced ferredoxin must be used to produce two moles of NADH using a ferredoxin-NAD^+^ reductase. Furthermore, as *C. acetobutylicum* does not have an oxidative pentose-phosphate pathway^26^ and as the nonphosphorylating NADPH-producing glyceraldehyde-3-P dehydrogenase encoded by *gapN* is expressed at a low level^19^, for each mole of *n*-butanol produced, one mole of reduced ferredoxin must also be used to produce one mole of NADPH using a ferredoxin-NADP^+^ reductase.

From this analysis, it is clear that ferredoxin-NAD^+^ and ferredoxin-NADP^+^ reductases are key to providing electrons for the production of *n*-butanol (Fig. 2), but until now, the proteins involved remained totally unknown. We, therefore, decided to purify all the enzymes with ferredoxin-NAD^+^ or ferredoxin-NADP^+^ reductase activity.

### Purification and characterization of the ferredoxin-NADP^+^ reductase of *C. acetobutylicum*

Proteins with ferredoxin-NADP^+^ reductase activities were purified under strict anaerobic conditions from *C. acetobutylicum* ATCC 824 crude extract as described in the methods. Proteins were first captured using a Capto-DEAE matrix, and active eluted fractions were pooled and then purified using a Resource Q column. After concentration, active eluted fractions were finally loaded onto a Superose 12 column. The results of a traditional purification are presented in Table 1. Activities were measured at 340 nm by NADP^+^ reduction using CA_C0303 reduced ferredoxin as the electron donor. During the purification process, the purified enzyme lost 60% of its activity after 48 h.

**Table 1:** Purification of ferredoxin-NADP^+^ reductases from *C. acetobutylicum*

Steps	Activity (units)	Protein (mg)	Specific activity (units/mg)	Recovery (%)	Purification fold
Crude extract	2.7	150	0.02	100	1
Streptomycin sulfate supernatant	2.46	104	0.02	92	1.33
CAPTO-DEAE column	0.52	1.93	0.27	20	15
Resource Q column	0.066	0.22	0.3	2.44	12.5
Superose 12 column	0.02	0.012	1.73	0.74	96

Active eluted fractions from gel filtration were then subjected to denaturing gel electrophoresis. As shown in Supplementary Fig. 1, the increase in ferredoxin-NADP^+^ reductase activity seems to be linked to the concentration of a protein with an apparent molecular mass of 45 kDa on SDS-PAGE.

**Fig. 3:**
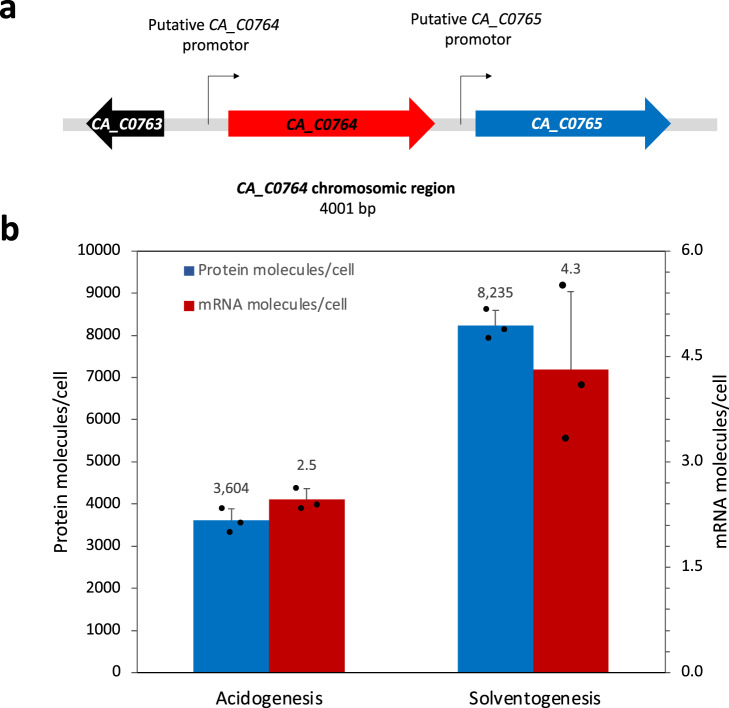
Transcriptional regulation of *CA_C0764*. *CA_C0764* chromosomic region of *C. acetobutylicum* (**a**) with putative -35 and −10 sequences of *CA_C0764* and *CA_C0765* analyzed with the BPROM tool. **b** Analysis of *CA_C0764* expression under acidogenesis and solventogenesis. Each error bar indicates the standard deviation around the mean of three biological samples from chemostat cultures. Data were extracted from the supplementary materials data from ref. 19. Source data are provided as a Source Data file.

The region of the gel corresponding to this protein was eluted, digested by trypsin and analyzed by nano-LC–MS/MS as described in the methods. The encoding gene is *CA_C0764*, annotated as an NADPH-dependent glutamate synthase beta subunit. Identical treatments were applied to the 40 and 55 kDa proteins, which were identified as thiolase (*CA_C2873*) and phosphoribosylaminoimidazole carboxamide formyltransferase-IMP cyclohydrolase (*CA_C1395*). None of these proteins are oxidoreductases. The gene identification results were used to extract the quantitative transcriptomic and proteomic data performed^19^, and it was confirmed that the expression of *CA_C0764* from a monocistronic operon was higher under solventogenic condition than under acidogenic condition (Fig. 3).

To validate the ferredoxin-NADP^+^ reductase activity of the CAC0764 protein, the *CA_C076*4 gene was cloned into a replicative plasmid to be fused with a small tag (Strep-tag II) placed in the C-terminal position of CAC0764, as described in Supplementary Method 1. The recombinant protein was homologously overexpressed in MGC*Δcac1502*^27^ (Supplementary Method 2) and then purified from the crude extract in a single step using affinity chromatography on a Strep-Tactin column. Recombinant proteins were eluted with desthiobiotin, and the purity of the eluted fraction was checked by SDS-PAGE with Coomassie blue staining (Supplementary Fig. 2).

**Fig. 4:**
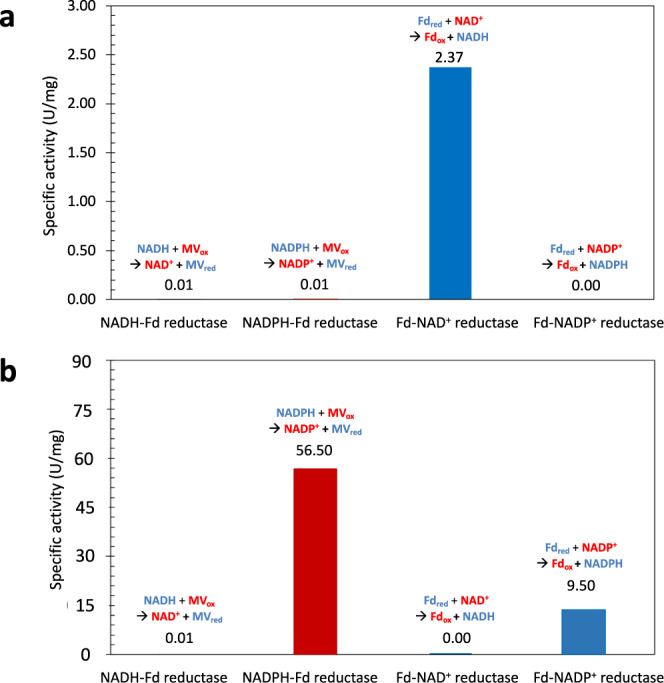
Enzyme activities of the purified ferredoxin-NAD(P)^+^ reductases. **a** CAC0764-Strep-tag and **b** Bcd–EtfB-Strep-tag-EtfA complex overexpressed and purified from *C. acetobutylicum* cells. Enzymatic activities were determined using oxidized methyl viologen (red) or reduced ferredoxin (blue) according to the Methods section. Source data are provided as a Source Data file.

As expected, denaturing gel electrophoresis showed a single band in the eluted fraction corresponding to an apparent molecular mass of 45 kDa, demonstrating that CAC0764 was pure. In vitro ferredoxin-NAD(P)^+^ reductase and NAD(P)H-ferredoxin reductase activities were evaluated in the recovered pure fraction using NADP^+^ reduction with reduced ferredoxin as an electron donor or methyl viologen reduction by NADPH. According to Fig. 4a, purified CAC0764-Strep-tag II exhibited both NADPH-ferredoxin reductase and ferredoxin-NADP^+^ reductase activities, and neither activity was observed in the presence of NADH and NAD^+^. These results confirmed that CAC0764 is an FNOR enzyme that is strictly NADPH/NADP^+^ dependent. CAC0764 shares sequence homology (~40% identity) with one subunit, NfnB, of the NADH-dependent ferredoxin-NADP^+^ oxidoreductase (NfnAB) from *Clostridium kluyveri*. NfnAB is an electron bifurcating enzyme that catalyzes the endergonic reduction of NADP^+^ with NADH coupled to the exergonic reduction of NADP^+^ with reduced ferredoxin. In order to investigate if CAC0764 could catalyze the Nfn bifurcating reaction, an NAD^+^ reduction assay with purified CAC0764 in the presence of both NADPH and Fd_ox_ generating systems (see methods section) was performed but no activity could be detected demonstrating the CAC0764 is a true ferredoxin-NADP^+^ oxidoreductase.

Homologs of CAC0764 were found in all acetone–butanol–ethanol (ABE) producing Clostridia. For all the clostridia belonging to clade I^27^, *C. acetobutylicum, C. roseum/C. aurantibutyricum/C. felsineum, and a C. pasteurianum*, the CAC0764 homologs were expressed as a monocistronic operon while for the Clostridia of clade II, *C. saccharobutylicum, C. beijerinckii, C. diolis* DSM 15410, *C. pasteurianum* NRRL B-598*,* and *C. saccharoperbutylacetonicum*^27^ they were expressed as a bicistronic operon with a gene homologous to *nfnA* from *C. kluyveri*.

### Purification and characterization of the ferredoxin-NAD^+^ reductase of *C. acetobutylicum*

Proteins with ferredoxin-NAD^+^ reductase activities were purified under strict anaerobic conditions from *C. acetobutylicum* ATCC 824 crude extract as described in the methods. Proteins were first captured using a Capto-DEAE matrix and eluted with a linear gradient of NaCl from 0.1 to 0.25 M, yielding two peaks (one minor and one major) of ferredoxin-NAD^+^ reductase activities. The peak activity eluted fractions were pooled separately and then concentrated before being finally loaded onto a Superose 12 or a Resource Q column. The results are presented in Table 2.

**Table 2:** purification of ferredoxin-NAD^+^ reductases from *C. acetobutylicum*

Steps	Eluted peak	Activity (units)	Protein (mg)	Specific activity (units/mg)	Recovery (%)	Purification fold
Crude extract		13.4	203	0.07	100	1
Streptomycin sulfate supernatant		9.02	102	0.09	67	1.28
Capto-DEAE column	Major peak 1	7.3	5.44	1.34	54	19.14
	Minor peak 2	1.27	4,06	0.31	9.5	4.49
Peak 1 on Superose 12 column		3.43	1.5	2.29	25	32.7
Peak 2 on Resource Q column		0.56	1.5	0.40	4.2	5.71

Active eluted fractions from gel filtration and Resource Q were then loaded onto denaturing gel electrophoresis. As shown in Supplementary Fig. 3, ferredoxin-NAD^+^ reductase activity from gel filtration was associated with the presence of 3 proteins of 41, 37, and 34 kDa, and ferredoxin-NAD^+^ reductase activity from Resource Q was linked to the presence of two proteins of 167 and 53 kDa. All proteins were eluted, digested by trypsin and analyzed by nano-LC–MS/MS as described in Methods. The results indicated that the three proteins eluted from gel filtration were the three subunits of butyryl-CoA dehydrogenase encoded by *bcd*, *etfB* and *etfA*, and the two proteins eluted from Resource Q were the two subunits of NADH-dependent glutamate synthase encoded by *gltA* and *gltB*. The BCD enzyme complex was previously shown to have NADH-ferredoxin reductase activity in the presence of crotonyl-CoA^19^. This study shows clearly that BCD also has ferredoxin-NAD^+^ reductase activity in the absence of crotonyl-CoA or butyryl-CoA.

To determine if ferredoxin-NAD^+^ reductase activity could be obtained in the absence of Bcd, the *etfB-etfA* genes were cloned on a replicative plasmid with a sequence encoding Strep-tag II placed in the 3’ position of *etfB* with and without *bcd* as the first gene of the synthetic operon^19^. The recombinant proteins were produced in *E. coli* from the two plasmids and then purified on a Strep-Tactin column. When all three genes were expressed, an active complex could be purified, while when only two genes were expressed, only EtfB could be purified, indicating that EtfB and EtfA cannot form a complex in the absence of Bcd.

The ferredoxin-NAD^+^ reductase, ferredoxin-NADP^+^ reductase, NADH-ferredoxin, and NADPH-ferredoxin reductase activities of the Bcd–EtfB-EtfA complex were evaluated in the purified active fraction recovered from *C. acetobutylicum*. According to Fig. 4b, the purified complex exhibited ferredoxin-NAD^+^ reductase activity, but no ferredoxin-NADP^+^ reductase activity, no NADH-ferredoxin, and no NADPH-ferredoxin reductase activity were detected. These results confirmed that in addition to its butyryl-CoA dehydrogenase activity, the Bcd–EtfB-EtfA complex can exhibit ferredoxin-NAD^+^ reductase activity in the absence of any CoA derivative.

Finally, the ferredoxin-NAD^+^ reductase, ferredoxin-NADP^+^ reductase, NADH-ferredoxin reductase, and NADPH-ferredoxin reductase activities of the GltAB complex were evaluated in the purified active fraction recovered from *C. acetobutylicum*. Both ferredoxin-NAD^+^ reductase and NADH-ferredoxin reductase activities were detected, while very low ferredoxin-NADP^+^ reductase or NADPH-ferredoxin reductase activity was measured, indicating that GltAB is mainly NADH/NAD^+^ dependent (Supplementary Fig. 4).

### Construction of knockout mutants of the genes encoding enzymes with ferredoxin-NAD^+^ reductase and ferredoxin-NADP^+^ reductase activities

**Fig. 5:**
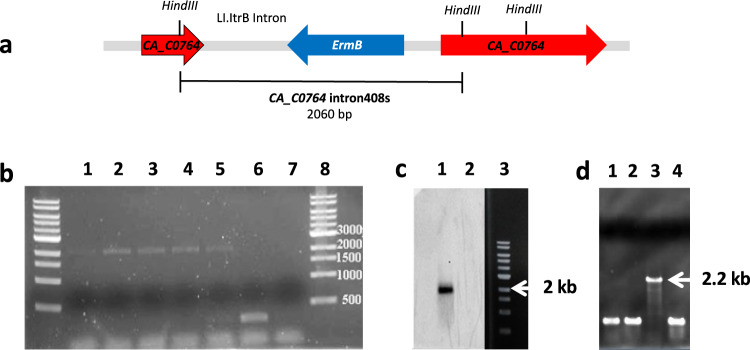
Construction of the *MGCΔcac1502-CA_C0764-408s::CT* and *MGCΔcac1502-gltb181s::CT* mutants. **a** Schematic representation of the *CA_C0764* gene with a group II intron inserted at position 408 on the sense strand of *CA_C0764*. **b** PCR screening for the identification of putative *CA_C0764-408s::CT* mutants using gene-specific primers flanking the intron insertion site (lanes 2–5) (expected size 2060 bp) and PCR control with wild-type DNA (lane 7) (expected size 270 bp), lanes 1 and 8 DNA ladder. **c** Southern hybridization to demonstrate the presence of a single intron insertion in the selected *MGCΔcac1502cac0764-408s::CT* mutant. The intron probe was DIG-labeled and hybridized to HindIII-HF digested genomic DNA of the *MGCΔcac1502cac0764-408s::CT* mutant (lane 2) with an expected size of 1970 bp. The HindIII-HF digested genomic DNA of the *C. acetobutylicum MGCΔcac1502* strain (lane 1) was also tested as a negative control. Lane 3 is a 1 kb DNA ladder. **d** PCR screening for the identification of one putative *gltb181s::CT* mutant (among three colonies that were tested) using gene-specific primers flanking the intron insertion site (lanes 1–3) (expected size 2200 bp) and PCR control with wild-type DNA (lane 4) (expected size 500 bp), lane 1 DNA ladder. Source data are provided as a Source Data file.

To investigate the role of ferredoxin-NAD(P)^+^ reductases in the production of butanol in vivo, group II intron-based ClosTron technology^28^ was used to inactivate the *CA_C0764, gltB* and *etfB* genes in the *MGCΔcac1502* a useful mutant for functional genomics analysis^29^. This technology uses the insertion of a group II intron into a genomic target site coupled to a retrotransposition-activated marker (erythromycin resistance), allowing stable gene inactivation. The retargeted introns were first directed to be inserted at position 407/408 on the sense strand of *CA_C0764* and at position 181/182 on the sense strand of *gltB* (Supplementary Method 1). After mutagenesis, the insertion mutants were checked by combining PCR screening, sequencing and Southern hybridization (Fig. 5) (Supplementary Method 3). Both *MGCΔcac1502-CA_C0764-408s::CT* and *MGCΔcac1502-gltb181s::CT* were successfully constructed following the procedure described in Supplementary Method 2.

A similar approach using the ClosTron method was used to inactivate the *etfB* gene. Despite repeated attempts and the use of at least two different retargeted ClosTron plasmids, insertions into the *etfB* gene could not be obtained. The low number of erythromycin-resistant clones that did arise had apparently inserted elsewhere in the genome, suggesting that the ferredoxin-NAD^+^ reductase activity of BCD is essential for *C. acetobutylicum*.

### Role of ferredoxin-NADP^+^ reductase in the central metabolism of *C. acetobutylicum*

**Fig. 6:**
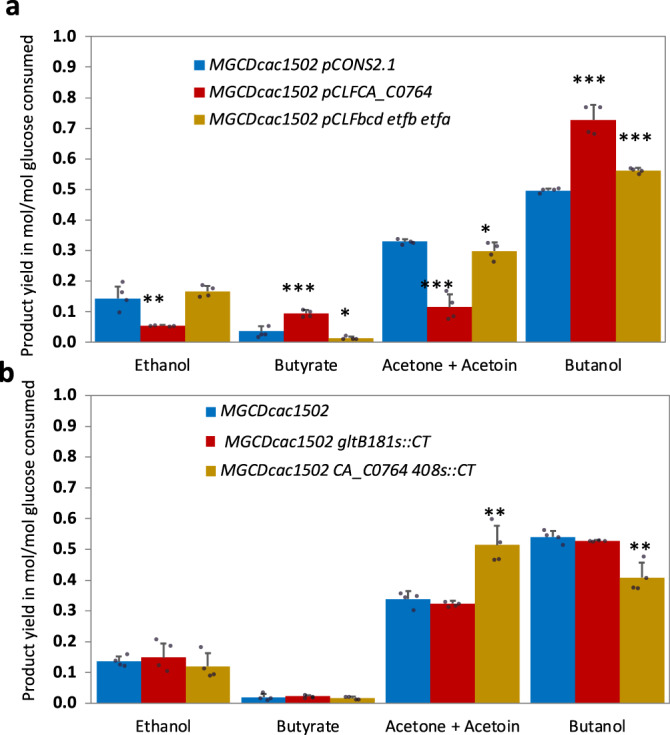
Comparative final product yields in mol/mol of glucose consumed for all *C. acetobutylicum* strains. **a** *MGCΔcac1502 (pCons2-1)* (blue), *MGCΔcac1502 (pCLFCA_C0764)* (red), *MGCΔcac1502 (pCLFbcd-etfb-etfa*) (brown). **b** *MGCΔcac1502* (blue), *MGCΔcac1502-gltB181s::CT* (red), *MGCΔcac1502-CA_C0764-408s::CT* (brown). Each error bar indicates the standard deviation around the mean of four independent cultures in serum bottles at 37 °C in SM medium^7^. Asterisk indicates that the results were significantly different from the control according to the one-sided Student’s *t* test, with *P value ≤ 0.05, **P value ≤ 0.01, and ***P value ≤ 0.001. Source data are provided as a Source Data file.

To better understand the role of ferredoxin-NADP^+^ reductase in the central metabolism of *C. acetobutylicum*, the growth and product formation of a strain with an inactivated cac0764 gene (*MGCΔcac1502CA_C0764-408s::CT*) and a strain overexpressing *cac0764* (*MGCΔcac1502 (pCLFCA_C0764)*) (Supplementary Methods 1 and 2) were compared to the control *MGCΔcac1502* strain and the *MGCΔcac1502 (pCons2-1*) strain containing an empty control plasmid, respectively. The final product yields of all strains after 10 days of culture are shown in Fig. 6a, b.

**Fig. 7:**
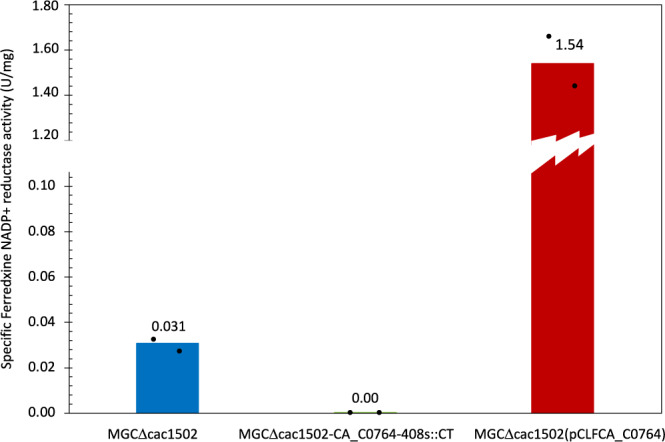
Specific ferredoxin-NADP^+^ reductase activity in cell-free extracts. MGCΔcac1502 (blue) was used as a control strain and compared to both the *MGCΔ1502-CA_C0764-408s::CT* (green) and *MGCΔcac1502(pCLFCA_0764)* (red) mutants. Bar graphs are averages from two biological duplicates. For the enzyme assays, see “Methods”. Source data are provided as a Source Data file.

Figure 6b shows that the inactivation of the gene encoding the ferredoxin-NADP^+^ reductase caused a marked decrease in the butanol yield (40% of the theoretical yield) in comparison to the parental *MGCΔcac1502* strain (54% of the theoretical yield). Conversely, the acetone + acetoin yield was almost doubled (34% of the theoretical yield in the parental *MGCΔcac1502* strain versus 50% of the theoretical yield when *CA_C0764* was inactivated). On the other hand, the overexpression of the ferredoxin-NADP^+^ reductase-encoding gene (Fig. 6a) favored butanol production and significantly increased the butanol yield (from 50% to 72% of the theoretical yield) at the expense of acetone + acetoin production, whose yield was strongly reduced from 33% to 11% of the theoretical yield. Finally, the strain with the ferredoxin-NADP^+^ reductase-encoding gene inactivated could be complemented by the introduction of the *pCLF0764* plasmid overexpressing *CA_C0764*, which restored high butanol production (68% of the theoretical yield) (Supplementary Fig. 5). All these results were confirmed by ferredoxin-NADP^+^ reductase activity measurements (Fig. 7), which showed negligible ferredoxin-NADP^+^ reductase activity (<0.001 U/mg) when *CA_0764* was inactivated and high ferredoxin-NADP^+^ reductase specific activity when *CA_0764* was overexpressed from the *pCLF0764* plasmid (1.54 U/mg + /− 0.07, versus 0.0326 U/mg + /− 0.007 for the control).

These results clearly demonstrated the involvement of the ferredoxin-NADP^+^ reductase enzyme in the butanol production pathway of *C. acetobutylicum* under solventogenic conditions. Moreover, it was also demonstrated that the yield of *n*-butanol was limited by the level of ferredoxin-NADP^+^ reductase activity.

### Role of each of the two enzymes with ferredoxin-NAD^+^ reductase activity in the central metabolism of *C. acetobutylicum*

To better understand the role of ferredoxin-NAD^+^ reductases in the central metabolism of *C. acetobutylicum*, a strain with an inactivated *gltB* gene ***(****MGCΔcac1502-gltB181s:CT)* and a strain overexpressing the genes encoding the Bcd–EtfB-EtfA complex *(MGCΔcac1502 (pCLF bcd-etfb-etfa))* (Supplementary Methods 1 and 2) were grown anaerobically in liquid flasks containing SM with 60 g/l glucose under the same conditions, and the growth and product formation were measured. Comparative phenotypic analysis was performed by measuring both glucose consumption and the concentration of fermentation products (Fig. 6). Inactivation of *gltB* had no effect on the product profile suggesting that the GltAB enzyme complex plays a minor role in the production of NADH from reduced ferredoxin. In contrast, overexpression of the *bcd-etfB-etfA* operon increased the butanol yield (from 50 to 56% of the theoretical yield) at the expense of acetone and acetoin production, which was reduced from 33 to 28% of the theoretical yield. This plus the fact that a knockout *etfB* mutant is not viable, strongly suggests that the BCD complex is responsible for the ferredoxin-NAD^+^ reductase activity in *C. acetobutylicum*.

### NADPH production in the *MGCΔ1502-CA_C0764-408s::CT* mutant

**Fig. 8:**
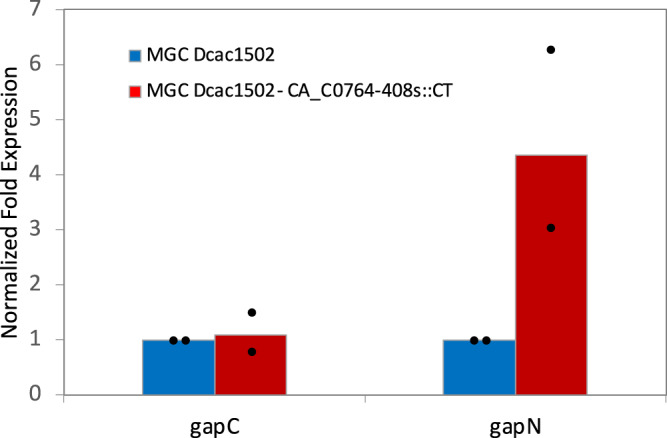
Normalized fold expression of *gapC and gapN* genes. *MGCΔcac1502* (blue) was used as a control strain and compared to the *MGCΔcac1502-CA_C0764-408s::CT* (red) mutant. Bar graphs are averages from two biological duplicates. The expression of *gapC* and *gapN* was measured using RTq-PCR and the *fabZ* gene (*CA_C3571*) as an internal standard according to Supplementary Method 4. For each biological sample, the average of the technical triplicates is represented (black circle). Source data are provided as a Source Data file.

In this study, we showed that under solventogenic conditions, CAC0764 is the sole enzyme responsible for ferredoxin-NADP^+^ reductase activity in *C. acetobutylicum*. As it was previously demonstrated that the oxidative pentose-phosphate pathway is missing in *C. acetobutylicum*^21,26^, we addressed the following question: how can the *MGCΔcac1502-CA_C0764-408s::CT* mutant generate the NADPH needed for residual butanol production and anabolic reactions? Another NADPH-producing enzyme already identified in *C. acetobutylicum* is glyceraldehyde-3-phosphate dehydrogenase (GapN), encoded by *CA_C3637*, a nonphosphorylating enzyme that catalyzes the oxidation of glyceraldehyde-3-phosphate to 3-phosphoglycerate (Fig. 1). In solventogenic chemostat cultures, this enzyme was shown to catalyze less than 5% of the total flux of the EMP pathway^19^. To determine whether *gapN* could be upregulated in the *MGCΔcac1502-CA_C0764-408s::CT* mutant in comparison to the *MGCΔ1502* control strain, quantitative reverse-transcriptase PCR (RT-qPCR) analysis was carried out in both strains to determine the relative expression of the *gapN* and *gapC* (encoding the NADH-dependent GAPDH) genes using the *fabZ* gene as the normalization reference gene^30^. Experiments were performed as described in Supplementary Method 4, and the normalized fold expression of both *gapN* and *gapC* in both the *MGCΔcac1502* and *MGCΔcac1502-CA_C0764-408s::CT* strains is presented in Fig. 8.

According to Fig. 8, the relative expression level of *gapN* in the *MGCΔ1502-CA_C0764-408s::CT* mutant was 4.3-fold higher than the relative expression level of *gapN* in *MGCΔcac1502*, showing that this gene was upregulated in the mutant strain.

Based on the phenotypic analysis performed under solventogenic conditions, the ferredoxin-NADP^+^ reductase activity determination in the cell crude extract, and the GapN expression results, a redox analysis was performed to determine how reduced ferredoxin is used to achieve the redox balance in each strain (Supplementary Table 3).

When CAC0764 is inactivated, reduced ferredoxin is mainly used for hydrogen production by HydA hydrogenase, a lower amount is used for NADH production (needed for ethanol, butyrate, lactate, and butanol synthesis), and 28% of the EMP flux is catalyzed by GapN to produce the NADPH needed for *n-*butanol synthesis and anabolism. In contrast, when CAC0764 is overexpressed, reduced ferredoxin is mainly used for the NADH and NADPH formation needed for ethanol, butyrate, and butanol production and anabolic reactions.

## Discussion

Ferredoxin-NAD^+^ and ferredoxin-NADP^+^ reductase activities were measured in *C. acetobutylicum* more than 40 years ago^20^. Their activities under different physiological conditions were further studied by other groups, but the proteins were never purified and characterized^2,6^. Their key role in butanol production was also suggested long ago from a stoichiometric model of the metabolism^31^ but never demonstrated using a reverse genetic approach. More recently, using an updated genome-scale model constrained by transcriptomics and proteomics data^19^, the study quantified and demonstrated the need for both ferredoxin-NAD^+^ and ferredoxin-NADP^+^ reductases to produce butanol under solventogenic conditions. As all the efforts to identify these proteins through blast searches were unsuccessful, a classical purification protocol was developed to isolate both the ferredoxin-NAD^+^ and ferredoxin-NADP^+^ reductases of *C. acetobutylicum*. The applied strategy enabled the identification of only one protein catalyzing ferredoxin-NADP^+^ reductase activity (encoded by the *CA_C0764* gene) and two enzyme complexes (Bcd–EtfB-EtfA and GltAB) having a ferredoxin-NAD^+^ reductase activity.

The ferredoxin-NADP^+^ reductase from *C. acetobutylicum* does not share any amino acid identity with previously described ferredoxin-NADP^+^ reductases from other bacteria and shares 30% identity with GltB (the β chain of NADH-dependent glutamate synthase) (Supplementary Fig. 6), explaining its incorrect annotation. However, it is expressed as a monocistronic operon, which does not fit with a genetic organization as a bicistronic operon of glutamate synthase-encoding genes. CAC0764 was demonstrated to be strictly NADPH/NADP^+^ dependent, and FAD is required to retain full enzyme activity, as is generally described for the ferredoxin-NADP^+^ reductase enzyme family^22^. Homologs of CAC0764 were found in all ABE (acetone–butanol ethanol) producing Clostridia. For all the clostridia belonging to clade I^27^, *C. acetobutylicum, C. roseum/C. aurantibutyricum/C. felsineum*, and a *C. pasteurianum* the CAC0764 homologs were expressed as a monocistronic operon while for the Clostridia of clade II, *C. saccharobutylicum, C. beijerinckii, C. diolis* DSM 15410, *C. pasteurianum* NRRL B-598*, and C. saccharoperbutylacetonicum*^27^ they were expressed as a bicistronic operon with a gene homologous to *nfnA* from *C. kluyveri*. We can then conclude that for all the clade I solventogenic Clostridia, NADPH is produced by a ferredoxin-NADP^+^ oxidoreductase while for all the clade II Clostridia, NADPH is produced by a NADH-dependent ferredoxin-NADP^+^ oxidoreductase.

The two enzyme complexes with NADH-ferredoxin reductase activity BCD and GltAB were previously shown to be a butyryl-CoA dehydrogenase^19^, and an NADH-dependent glutamate synthase^19^, respectively. As the inactivation of *gltB* had no effect on the product profile, the GltAB enzyme complex probably plays a minor role in vivo in electrons transfer from reduced ferredoxin to NAD^+^. In contrast, the fact that (i) overexpression of the *bcd-etfB-etfA* operon increased the butanol yield and (ii) a knockout *etfB* mutant is not viable, strongly suggests that the BCD complex is responsible for the ferredoxin-NAD^+^ reductase activity in *C. acetobutylicum*. BCD is a bifurcating enzyme reducing crotonyl-CoA to butyryl-CoA with the consumption of two NADHs and the production of one reduced ferredoxin. This allows the endergonic transfer of electrons from NADH to oxidize ferredoxin and eliminate the excess NADH associated with acetate production in acidogenic conditions^6,19^. What is demonstrated here is that in the absence of crotonyl-CoA or butyryl-CoA, this enzyme can also carry exergonic electrons transfer from reduced ferredoxin to NAD^+^.

From a physiological perspective, inactivation of the ferredoxin-NADP^+^-encoding gene significantly decreased butanol production and increased acetone production, while its overexpression had the opposite effect, with a very high *n*-butanol yield from glucose of 72% of the theoretical value (Supplementary Fig. 7). This suggests that butanol production, under solventogenic conditions, is potentially limited by the flux of NADPH production and demonstrates that the carbon fluxes can also be modulated by manipulating the electron fluxes. Furthermore, in the absence of ferredoxin-NADP^+^ reductase, *C. acetobutylicum* maintains a certain flux of NADPH production by expressing a higher level of *gapN*, a gene coding for nonphosphorylating NADP^+^-dependent glyceraldehyde-3-P dehydrogenase. In this mutant, up to 28% of the EMP flux is catalyzed by GapN, resulting in lower ATP production.

Attempts to knock out *etfB* to abolish ferredoxin-NAD^+^ reductase activity have been unsuccessful. This suggests that *C. acetobutylicum* cannot grow in the absence of ferredoxin-NAD^+^ reductase activity. Consistently, all the electron flux analyses performed on chemostat cultures of *C. acetobutylicum* in acidogenic, solventogenic or alcohologenic conditions^19^ show a high flux in the reaction catalyzed by ferredoxin-NAD^+^ reductase. Furthermore, we can demonstrate using the *i*Cac967 genome-scale model that an *etfB* mutant that would be unable to produce butyrate and butanol and would have no ferredoxin-NAD^+^ reductase activity could survive only if it produced lactate as the only fermentation product. Such a drastic redirection of the metabolic fluxes is probably not possible by regulation of the metabolic pathway and might explain why the *etfB* mutant is not viable.

The discovery of the ferredoxin-NADP^+^ and ferredoxin-NAD^+^ reductase-encoding genes and the demonstration of their key role in butanol production present the possibility of complementary metabolic engineering strategies to create a homobutanologenic *C. acetobutylicum* strain.

## Methods

### Bacterial strains, plasmids, and oligonucleotides

All bacterial strains, plasmids, and oligonucleotides used in or derived from this study are listed in Supplementary Tables 1 and 2. Procedures for plasmid construction are detailed in Supplementary Method 1.

### Culture media and growth conditions

*E. coli* strains were grown aerobically at 37 °C in Luria–Bertani (LB) medium supplemented, when necessary, with ampicillin (100 µg/mL) and/or chloramphenicol (30 µg/mL). Agar (15 g/L) was added prior to sterilization on LB agar plates.

*MGCΔcac1502*, *MGCΔcac1502-CA_C0764-408s::CT*, and *MGCΔcac1502 gltB181s::CT* strains were kept in spore form at −20 °C in synthetic medium (SM). *MGCΔ1502-CA_C0764-408s::CT pCLFCA_C0764, MGCΔ1502 pCLFCA_C0764*, *MGCΔ1502 pCLF bcd-etfb-etfa* and *MGCΔ1502 pCons2-1* strains were kept on glucose SM plates with thiamphenicol (10 μg/mL) and directly used to inoculate liquid flask cultures containing glucose SM with thiamphenicol (50 μg/mL). The liquid flask cultures of all *C. acetobutylicum* strains were grown anaerobically at 37 °C in 30 mL of SM^7^ with 55 g/L glucose. Liquid cultures of the recombinant strains were run at least in quadruplicate.

### Analytical procedures

The cell concentration was measured turbidimetrically by monitoring the optical density (OD) at 620 nm; an experimentally derived correlation factor of 0.3 g cellular dry weight per OD_620 nm_ was used for the biomass concentration calculations. Glucose, pyruvate, lactate, acetate, butyrate, acetoin, glycerol, ethanol, acetone, and butanol concentrations were measured in the culture supernatants using high-performance liquid chromatography (HPLC) analysis (Agilent 1200 series, Massy, France)^17^. In all figures, yields were expressed in mol of each product forms per mol of glucose consumed. For strain comparison purposes, the butanol and acetone yield were also expressed in % of the theoretical yield with a theoretical yield of butanol or acetone formation of one mol of *n*-butanol or acetone per mol of glucose consumed.

### Enzyme assays

Ferredoxin-NAD(P)^+^ reductase activity and NAD(P)H-ferredoxin reductase activity assays were performed in an anaerobic workstation under a nitrogen atmosphere. All reagent solutions were prepared in assay buffer (previously boiled and degassed with nitrogen) and kept under a nitrogen atmosphere. Specific activities were determined in a range where linearity with protein concentration was established. Each enzyme assay was done at least in duplicate. One unit of enzyme activity is defined as the amount of enzyme that catalyzes the conversion of 1 µM of substrate per min. The concentrations of components in the reaction mixtures (1 mL of total volume) are given below.

In vitro ferredoxin-NAD(P)^+^ reductase activity was assayed by measuring the reduction of NAD^+^ or NADP^+^ using electrons from reduced ferredoxin (*CA_C0303*) with H_2_ as the reductant of ferredoxin (*CA_C0303*)^32^ in the presence of Fe–Fe hydrogenase from *Clostridium acetobutylicum* (*CA_C0028*)^33^. Ferredoxin (*CA_C0303*) was produced from anaerobic culture of the recombinant strain *E. coli* *ΔiscRpthl*-Fd-LL-C-Tag and purified in the in the form of Strep-tag II fused ferredoxin using a prepacked Strep-Trap HP Column (Cityva, Sweden) and an elution buffer containing 7.5 mM desthiobiotin^33^. The reaction was performed anaerobically at 37 °C in 100 mM Tris-HCl buffer (pH 7) with 2 mM DTT, 25 µM FAD, 13 µM ferredoxin or 150 µM methyl viologen, 1,6 mM NAD^+^ or NADP^+^, 6 U (or more) of purified hydrogenase HydA from *C. acetobutylicum* and crude extract (or purified protein), followed by monitoring the increase in A_340 nm_ as an indication of the appearance of NADH or NADPH using a spectrophotometer (Hewlett Packard 8453). After a gentle stream with hydrogen in the quartz cuvette cells, assays were initiated by the addition of ferredoxin and then, after the reduction of ferredoxin (~5 min), by the addition of NAD^+^ or NADP^+^. In all reactions, nonenzymatic rates were subtracted from the observed initial reaction rates.

In vitro NAD(P)H-ferredoxin reductase activity was assayed by monitoring the increase in A_560nm_ as an indication of the reduction of methyl viologen using a spectrophotometer (Hewlett Packard 8453). The reaction was carried out anaerobically at 37 °C in quartz cuvette cells in 100 mM Tris-HCl buffer (pH 7,6) with 2 mM DTT, 10 µM FAD, 250 µM NADPH, or NADH, ethanol 3% vol/vol, 45 U Adh (*S. cerevisiae*), 10 mM methyl viologen, and crude extract or purified protein. Assays were initiated by the addition of methyl viologen. In all reactions, nonenzymatic rates were subtracted from the observed initial reaction rates.

In vitro NADPH-dependent ferredoxin-NAD^+^ oxidoreductase activity was assayed^34^ by monitoring the increase in A_380 nm_ as an indication of the reduction of NAD^+^ using a spectrophotometer (Hewlett Packard 8453). The reaction was carried out anaerobically at 37 °C in quartz cuvette cells in 100 mM Tris-HCl buffer (pH 7,6) with 0.5 mM NADP^+^, 40 mM glucose-6-phosphate, and 2 U of glucose-6-phosphate dehydrogenase (NADPH regeneration system), 10 mM NAD^+^, 10 μM ferredoxin, and 1 U of purified HydA hydrogenase. N_2_ was used for the gas phase. The reaction was started with CAC0764 addition. (ε = 1.2 mM^−1^ cm^−1^). The formation of one μmol of NADH per min was defined as representing one unit.

The extinction coefficients of methyl viologen at 560 nm, of NADH at 380 nm and of NADH and NADPH at 340 nm were 7.71 mM^−1^ cm^−1^, 1.2 mM^−1^ cm^−1^, 6.22 mM^−1^ cm^−1^, and 6.29 mM^−1^ cm^−1^, respectively. The total protein concentration of the cell-free extract or purified fraction was determined using the Bradford method (Bio-Rad reagent)^35^ with bovine serum albumin as the standard.

### Purification of the ferredoxin-NAD^+^ and ferredoxin-NADP^+^ reductases in *C. acetobutylicum* under solventogenic conditions

The *C. acetobutylicum* ATCC 824 strain was kept in spore form at −20 °C in SM. The flask cultures of *C. acetobutylicum* strains were grown anaerobically in SM, inoculated with a spore stock at 10% (v/v), and heat-shocked at 80 °C for 15 min. Cells were grown at 37 °C to an OD_620 nm_ of ~2.0, and the pH was maintained by buffering the culture medium with calcium carbonate prior to inoculation of the bioreactor at 10% (v/v). pH-controlled batch fermentations were performed in SM. A 2 L Biostat B bioreactor (Sartorius, Aubagne, France) was used with a working volume of 1.3 L^36^. After sterilization, the medium was sparged with O_2_-free nitrogen for 30 min. During the course of the experiment, the medium was maintained under a slight nitrogen overpressure to avoid O_2_ entry into the reactor. All tubing was made of butyl rubber, and the reactor gas outlet was protected with a pyrogallol arrangement. Cultures were stirred at 300 rpm, the temperature was set at 35 °C, and the pH was maintained at 4.8 with the automatic addition of NH_4_OH (3 N). The cell concentration was measured turbidimetrically by monitoring the optical density (OD) at 620 nm (Biochrom libra S11), and product formation was measured in duplicate using HPLC analysis (Agilent 1200 series, Massy, France)^17^. When the OD_620 nm_ reached ~16, after the switch from the acidogenic to solventogenic phase, cells were harvested under hydrogen pressure and transferred into an anaerobic chamber. The cells were washed and concentrated 20 times in 100 mM Tris-HCl 2 mM DTT 10% glycerol (pH 7.6) buffer and frozen at −80 °C.

All purification procedures were performed under anaerobic conditions. All purification buffers were degassed in advance, and 10 µM FAD and 2 mM DTT were added to prevent nonreversible activity losses.

Frozen cells from solventogenic batch cultures of *C. acetobutylicum ATCC 824* were thawed and broken by sonication using an ultrasonic disintegrator (Vibracell 72434, Bioblock) at 4 °C in four cycles of 30 s at 2-min intervals. Debris was removed by centrifugation at 8600×*g* for 10 min at 4 °C (Sigma centrifuge 2–16 K). Nucleic acids were precipitated by the addition of streptomycin sulfate (200 μg/mL) to the supernatant and removed by centrifugation as described above. The recovered extract was then diluted five times in 100 mM Tris-HCl buffer (pH 8) before loading on a 5 mL HiTrap Capto-DEAE matrix (Cytiva, ref. 28-9165-40) connected to an AKTA purifier (Cytiva, Sweden). Active fractions were screened with the ferredoxin-NAD^+^ and ferredoxin-NADP^+^ reductase assay using reduced ferredoxin as an electron donor. The column was equilibrated in 100 mM Tris-HCl buffer (pH 8), and elution was performed with a 3-step gradient of 100 mM Tris-HCl + 1 M NaCl buffer (pH 8): 1 CV 0–4%, 20 CV 4–16% (target elution) and 5 CV 16–100%; 2 mL fractions were collected. For ferredoxin-NADP^+^ reductase activity, the most active fractions from the Capto-DEAE column were pooled before being loaded on a Resource Q column equilibrated in 100 mM Tris-HCl buffer (pH 8) for a second chromatographic step. The most active eluted fractions were then collected and concentrated on a Vivaspin 15/10,000 MW (Sartorius Stedim, ref. VS1502) to reduce the sample volume to 150 µL by centrifugation at 3000×*g* for 15 min. For the last purification step, a 150 μL sample volume was loaded on a Superose 12, 10/300 GL column (GE Healthcare, ref. 17-5173-01) previously equilibrated in 100 mM Tris-HCl + 150 mM NaCl buffer (pH 7.6), and 400 µL fractions were collected. For ferredoxin-NAD^+^ reductase activity, the active fractions from the Capto-DEAE column from each eluted peak were pooled before being loaded onto a Resource Q equilibrated in 100 mM Tris-HCl buffer (pH 8) for a second chromatographic step or onto a Superose 12 10/300 GL column (GE Healthcare, ref. 17-5173-01) previously equilibrated in 100 mM Tris-HCl + 150 mM NaCl buffer (pH 7.6), and 400 µL fractions were collected. Finally, the total protein concentration of the cell-free extract or purified fractions was determined using the Bradford method (Bio-Rad reagent)^35^ with bovine serum albumin as the standard.

The yields and purification factor of each step were calculated. The purity factor of the separate active fractions was also evaluated using SDS electrophoresis in 40-mL polyacrylamide gels.

### Identification of the gene coding for ferredoxin-NAD^+^ and ferredoxin-NADP^+^ reductase activities

Active eluted fractions collected after Superose 12 or Resource Q chromatography were loaded onto denaturing gel electrophoresis, and proteins were silver stained. For the ferredoxin-NADP^+^ reductase active fraction, the region of the gel corresponding to the protein at 45 kDa was used, and for the ferredoxin-NAD^+^ reductase active fractions, the regions of the gel corresponding to the proteins at (i) 41, 37, and 34 kDa and (ii) at 167 and 53 KDa were used. The gel regions were cut out using a sterile pipette tip. The gel plugs were then used for the identification of proteins by mass spectrometry. Each sample was subjected to trypsin digestion and analyzed by *nano-LC–MS/MS* on a CapLC-Q-TOF2 (Waters) and by MALDI on a MALDI MX (Waters). The candidate proteins were identified with ProteinLynx Global Server (Waters) and Mascot (Matrix Science) software using the Protein Data Bank entry for *C. acetobutylicum*. In both analyses, for ferredoxin-NADP^+^ reductase activity, only one protein was identified with a significant score (77% sequence coverage). For ferredoxin-NAD^+^ reductase activity, four proteins were identified with significant scores: (a) butyryl-CoA dehydrogenase, 58.6% sequence coverage; (b) EtfB, 78.4% sequence coverage; (c) GltA, 78.4% sequence coverage, and (d) GltB, 50.7% sequence coverage.

### Purification of CAC0764 and Bcd–EtfB–EtfA fused with a Strep-tag

CAC0764 protein and Bcd–EtfB–CST–EtfA complex protein were produced and purified under strict anaerobic conditions in the form of Strep-tag II fused proteins using a prepacked Strep-Trap HP Column (Cityva, Sweden). The desthiobiotin concentration in the elution buffer was 7.5 mM elution buffer, and 25 μM FAD was added to all buffers^33^.

### Reporting summary

Further information on research design is available in the Nature Research Reporting Summary linked to this article.

## Supplementary information

Supplementary Information

Reporting Summary

## Data Availability

The authors declare that all data supporting the findings of this study are available within the paper and its Supplementary Information file. Source data are provided with this paper.

## Source data

Source Data
