# Supplementary material for: Molecular characterization of the missing electron pathways for butanol synthesis in Clostridium acetobutylicum
Source: Nat Commun. 2022 Aug 10;13:4691. doi: 10.1038/s41467-022-32269-1 (PMC9365771; doi:10.1038/s41467-022-32269-1)
Supplement: Supplementary file 1 — Supplementary Information [file 41467_2022_32269_MOESM1_ESM.pdf]

Molecular characterization of the missing electron pathways for butanol synthesis in *Clostridium acetobutylicum*

Foulquier *et al.*

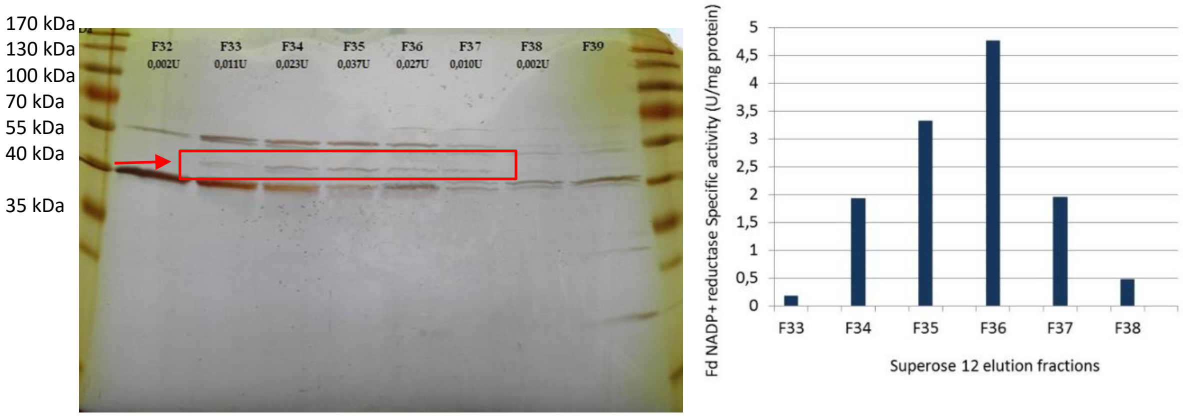

MW ~45 kDa

a **b**

Supplementary Fig. 1. Purification of the ferredoxin-NADP$^+$ reductase. This experiment was performed twice with similar results. a) SDS-PAGE analysis of active eluted fractions collected after Superose 12 chromatographic separation (proteins were silver stained). b) Ferredoxin-NADP$^+$ reductase activity of the corresponding fractions.

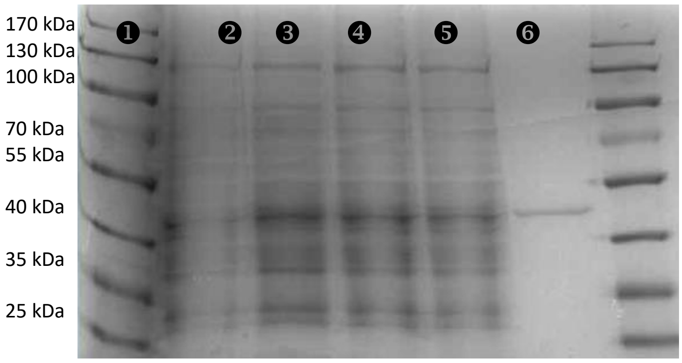

Supplementary Fig. 2. Purification of CAC0764-Strep-tag. This experiment was performed twice with similar results.SDS-PAGE of purified CAC0764-Strep-tag 1) protein ladder 2) cell-free extract 3) streptomycin sulfate treatment 4) avidin treatment 5) loaded sample 6) active eluted fraction. B) Enzymatic activities of the CAC0764-Strep-tag protein determined using methyl viologen (blue) or reduced ferredoxin (red).

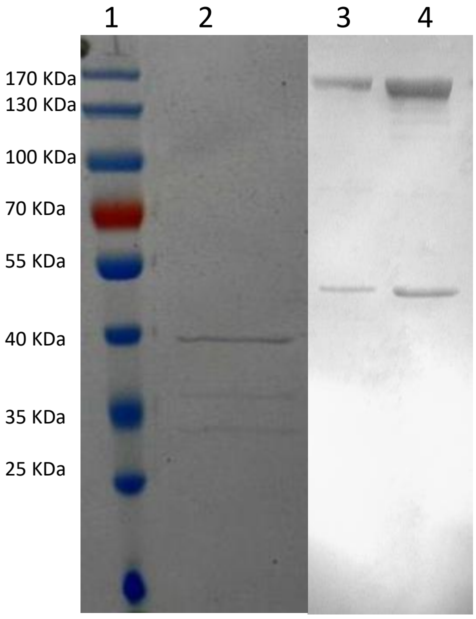

Supplementary Fig. 3. Purification of the ferredoxin-NAD$^+$ reductases. This experiment was only performed once. Silver-stained SDS-PAGE of the 1) protein ladder, 2) active eluted fractions from Resource Q, and 3-4) active eluted fraction from gel filtration.

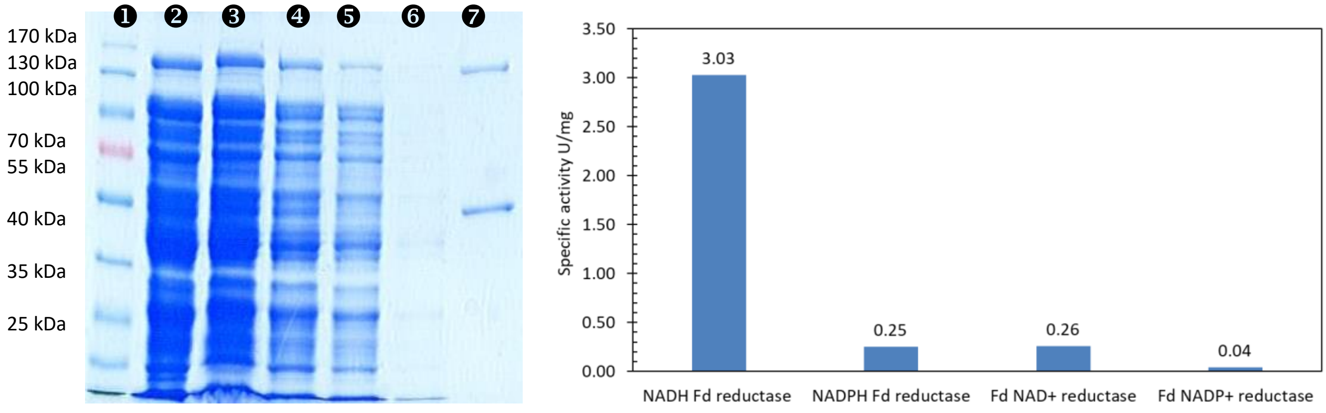

a **b**

Supplementary Fig. 4. Purification of the GltA-GltB-Strep-tag complex. This experiment was only performed once. **a)** Coomassie blue SDS-PAGE. 1) protein ladder 2) cell-free extract 3) streptomycin sulfate treatment 4) avidin treatment 5) loaded sample 6) washed fraction 7) active eluted fraction. **b)** Enzymatic activities of the purified GltA-GltB-Strep-tag complex determined using methyl viologen in all enzyme assays.

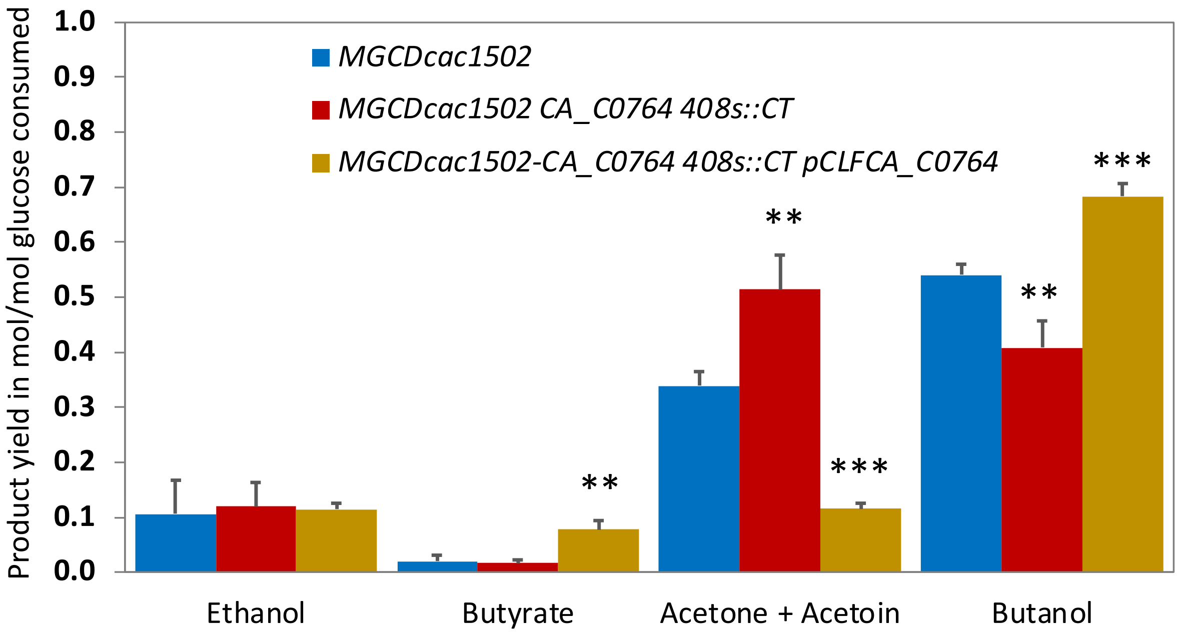

Supplementary Fig. 5. Comparative final product yields in mol/mol of glucose consumed for the three *C. acetobutylicum* strains: *MGCΔcac1502 (blue), MGCΔcac1502-CA_C0764-408s::CT (red), MGCΔcac1502-CA_C0764-408s::CT (pCLFCA_C0764) (brown).* Each error bar indicates the standard deviation around the mean of three independent cultures in serum bottles at 37°C in SM medium[8]. Asterisk indicates that the results were significantly different from the control according to the one-sided Student's *t*-test, with * for *P*-value ≤ 0.05, **for *P*-value ≤ 0.01, and ***for *P*-value ≤ 0.001. Source data are provided as a Source Data file.

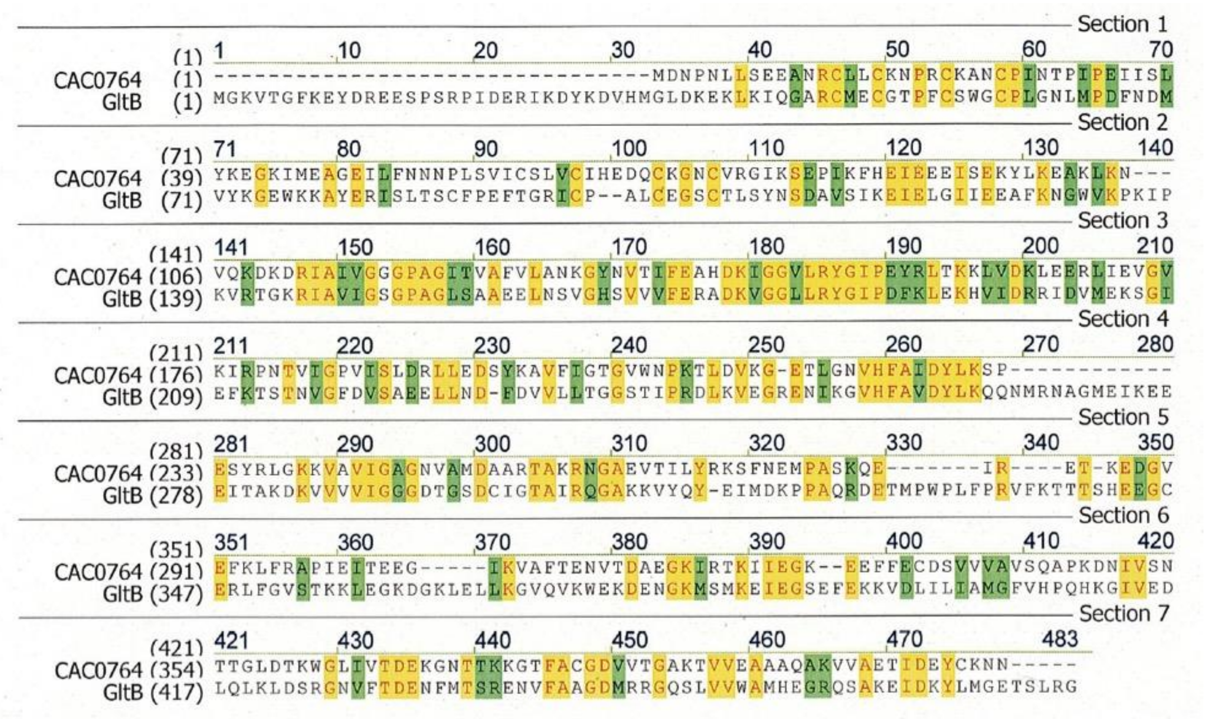

Supplementary Fig. 6. Alignment of the protein sequences of CAC0764 and GltB (CAC1674). Protein sequences of CAC0760 and GltB were aligned using AlignX from vector NTI (Invitrogen)

a

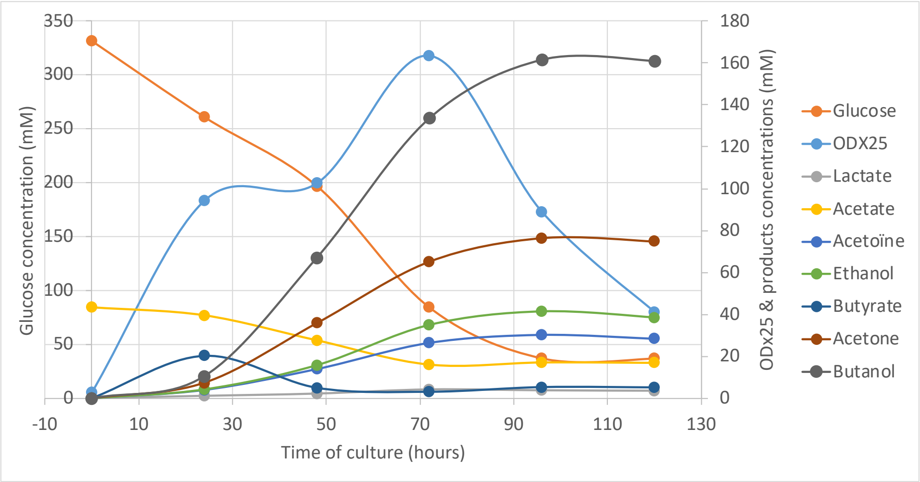

b

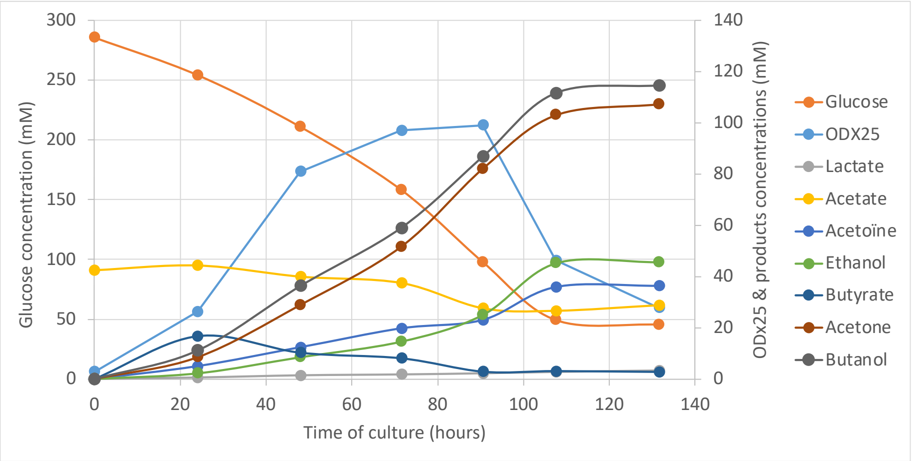

c

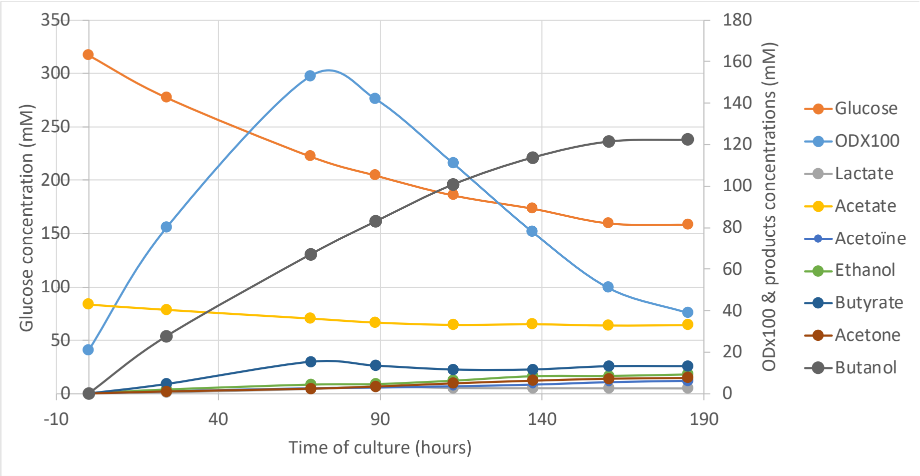

Supplementary Fig. 7. Batch fermentation of *C. acetobutylicum* strains: a) *MGCΔcac1502,* **b)** *MGCΔcac1502-CA_C0764-408s::CT* and **c)** *MGCΔcac1502 (pCLFCA_C0764).* Fermentations were performed in serum bottles at 37°C in SM medium[8].

Supplementary Table 1. List of strains and plasmids used in this study.

Strains/Plasmids	Relevant characteristics*	Source
C. acetobutylicum		
ATCC 824	Wild type	ATCC
MGC*Δ1502*	ΔCA_C1502	1
MGC*Δ1502 pCons2-1*	ΔCA_C1502	1
MGC*Δ1502-CA_C0764-408s::CT*	MGC*Δ1502 CA_C0764* mutant	This study
MGC*Δ1502-CA_C0764-408s::CT pCLFCA_C0764*	complemented MGC*Δ1502 CA_C0764* mutant	This study
MGC*Δ1502 pCLFCA_C0764*	overexpression of *CA_C0467* gene from the pCLF942 plasmid	This study
MGC*Δ1502-gltB181s::CT*	MGC*Δ1502 gltB* mutant	This study
MGC*Δ1502 pCLF bcd-etfB-etfA*	overexpression of *bcd-etfb-etfA* genes from the pCLF942 plasmid	This study
E. coli		
TOP10		Invitrogen
Plasmids		
pThl_HydA-LL-Ctag	MLS^{r}; AP^{r}; *repL*; *hydA* with hydA promoter and adc terminator	2
pCST-LL-CA_C0764	pPH_HydA-LL-Ctag derivative with *CA-C0764* insertion and pPH replacement with pthl	This study
pCST-LL-etfb-etfA	pPH_HydA-LL-Ctag derivative with etfb and etfa insertion and pPH replacement with pthl	3
pCST-LL-bcd-etfb-etfa	pCST-LL-etfBetfA derivative with bcd insertion	3
pSOS94	MLS^{r} ; AP^{r} ; repL ; ctfA ; ctfB ; adc with ptb promoter and adc terminator	GenBank: AY187685.1
pCLF1	Cm^{r} ; repL ; flp1	1
pCLF *CA_C0764*	pCLF1 derivative with *CA_C0764* with *ptb* promoter and *adc* terminator	This study
pCLF bcd-etfb-etfa	pCLF1 derivative with *bcd-etfb-etfa* with *ptb* promoter and *adc* terminator	This study
pMTL007		4
pMTL007::cac-CA_C0764-408s	ClosTron plasmid retargeted to *C. acetobutylicum CA_C0764* gene	This study
pMTL007::cac-gltB181s	ClosTron plasmid retargeted to *C. acetobutylicum gltB* gene	This study
pMTL007::cac-CA-C2710-159s	ClosTron plasmid retargeted to *C. acetobutylicum CA_C2710* gene	This study
pMTL007::cac-CA-C2710-101as	ClosTron plasmid retargeted to *C. acetobutylicum CA_C2710* gene	This study

*abbreviations: MLS^{r}: macrolide, lincosamide, Streptogramin B resistance; Ap^{r}: ampicillin resistance; catP: thiamphenicol resistance

Supplementary Table 2. List of primers used in this study (provided by Eurogentec UK).

Name	Oligonucleotide sequences (5’->3’)
408/409s-IBS	AAAAAAGCTTATAATTATCCTTAGGCTACAATGTTGTGCGCCCAGATAGGGTG
408/409s-EBS1d	CAGATTGTACAAATGTGGTGATAACAGATAAGTCAATGTTACTAACTTACCTTTCTTTGT
408/409s-EBS2	TGAACGCAAGTTTCTAATTTCGATTTAGCCTCGATAGAGGAAAGTGTCT
cac0764del_for	cgagccaataaaatttcacgagata
cac0764del_rv	ccaacctctataagtctttcttcaagctta
Ocac0764f	AGGATCCATCAAAATTTAGGAGGTTAGTTA
Ocac0764r	GGCGCCTTAATTATTCTTGCAATACTCATCAATAGTTTC
ESB universal	gtttactgaacgcaagtttctaatttcg
ErmB3’- R- F	cgccaaagtaaacaatttaagtaccgttac
fabZf	TCCAAGTATAGGCTTCTTTCCC
fabZr	GGTCATTACCCAGGTAAACCA
gapCf	CACATTAGATGGTCCACACAGAA
gapCr	AGCTAAGTCAGGAATAACTTGGC
gapNf	GGTTCTTGAGCTTGGTGGTAAA
gapNr	TTTACAGCAGTACACCTTTGGC
ccac0764BAMf	aattggatccatcaaaatttaggaggttagttagaatggataaccctaatttattgtcagaag
ccac0764SMAr	aattcccgggattattcttgcaatactcatcaatagtttc
CAC2710-101asIBS	aagcttataattatccttaccttcccttatagtgcgcccagatagggtg
CAC2710-159sIBS	gcacttgaggaagtgcgcccagatagggtg
CAC2710-159sEBS1d	acaaagaaaggtaagttagcttcctcgacttatctgttatcaccacatttgtacaatctg
RBS nat bcd_rec for	AAATGAGCACGTTAATCATTTAACATAGATAATTGGATCCAGGAGGTAAGTTTATATGGATTTTA<br>ATTTAACAAG
etfa_rec_rev	AGAAACAATCTCTTTTACTGGCAAATCATTAAGTGGCGCCTTAATTATTAGCAGCTTTAACTTGA<br>GCTATTAA
pCLF prom seq_for	AACACCACGTAGTTATTGGGAGG
pCLF term_rev	CTGCAAGAATGTGAGAGCTAG
psos prom univ	cttttggtcgtagagcacacgg
pSOSterm	ccgctcacaattccacacaacatacg
gltB_direct181S	GCTGCAGTTAATATGTAAGGGGGCGG
gltB_reverse181S	CCTTTGGAATCTTAGGCTTTACCCATCC

Supplementary Table 3. Comparative redox analysis of MGC*Δcac1502, MGCΔcac1502-CA_C0764-408s::CT an* *MGCΔcac1502 pCLFCA-C0764* strains cultured under solventogenic conditions.

	Strains		
	MGCΔ1502	*MGCΔ1502CA-C0764-408s::C*	*MGCΔ1502 pCLFcac0764*
mole of NADH produced in the glycolytic pathway	2	1.44	2
mole of NADPH produced in the glycolytic pathway	0	0.56	0
mole of NADH produced from reduced ferredoxin	0.55	0.47	1.6
mole of NADPH produced from reduced ferredoxin	0.74	0	0.8
mole of Fdred reoxidized by hydrogenase	1.2	1.85	0.33

The distribution of the electrons was described for 1 mole of glucose consumed in the glycolytic pathway.

Supplementary Method 1. plasmid construction

Construction of *pMTL007*::cac-*CA_C0764*-408s

The intron target site was identified at bp 408/409 (from the start of the ORF) on the sense strand, and the intron retargeting PCR primers 408/409-IBS, 408/409-IEBS1d, and 408/409-EBS2 (Supplementary Table 2) were designed using a computer algorithm[5]. The three primer sets 408/409-IBS, 408/409-IEBS1d, 408/409-EBS2 and the EBS universal primers were used in a single-tube reaction with the pMTL007 plasmid[4] to mutate the intron at several positions spanning a 350 bp region. PCR designed to retarget the intron by primer-mediated mutation was performed according to the protocol of the Targetron Gene Knockout System Kit (http://www.sigmaaldrich.com/life-science/functional-genomics-and-rnai/targetron.html).

The 350 bp PCR fragment was purified and then cloned into the pMTL007 plasmid at the HindIII and BsrGI sites to replace the original intron fragment using T4 DNA ligase (New England Biolabs, Evry, France). The ligation product was then introduced into Top10 chemically competent *E. coli* cells (Invitrogen™). Single colonies were then grown in LB liquid culture supplemented with ampicillin (100 µg/mL) overnight at 37°C to finally carry out DNA plasmid extraction (GenElute HP plasmid miniprep kit, Sigma) and check for the presence of the *pMTL007::cac-CA_0764-408s* plasmid. The retargeted *pMTL007::cac-CA_0764-408s* plasmid was finally controlled by restriction and by DNA sequencing using the 408/409-IBS and 408/409-IEBS1d primers (Supplementary Table 2).

Construction of *pMTL007Ca::gltB-180s*

A similar method was applied to generate the intron-retargeted *pMTL007Ca::gltB-180s* plasmid. The intron target site was identified at 181/182 bp on the sense strand using the Perutka algorithm[5].

Construction of *pMTL007Cs::2710-101as* and *pMTL007Cs::2710-159s*

The Perutka algorithm[5] was used to identify the intron target site. Two positions, 101/102 on the antisense strand and 159/160 on the sense strand, were selected.

Construction of *pCLF-CA_C0764*

The *CA_C0764* gene was amplified from the genomic DNA of *C. acetobutylicum ATCC824* using the Ocac0764f and Ocac0764r primers (Supplementary Table 2). The primers were designed to introduce an RBS region along with the *CA_C0764* gene, as well as placing BamHI and SfoI restriction sites upstream and downstream, respectively. The amplified PCR fragment using the proofreading Phusion DNA polymerase (New England Bioloabs) was then subcloned into a Zero Blunt TOPO vector (Invitrogen, Saint Aubin, France) to yield the Zero Blunt TOPO–*CA_C0764* plasmid, and the product was sequenced using universal primers T7P and T3P to assure that no mutations were introduced. The fragment containing the *CA_C0764* gene was purified on an agarose gel after digestion of the Zero Blunt TOPO–*cac0764* vector with BamHI and SfoI. The 7 kb pSOS94 vector[6] was also digested with BamHI and SfoI (New England Biolabs) and ligated to the BamHI-SfoI digested *sadh* gene, yielding the 6.25 kb pSOS94-CA-C0764 vector. The *pSOS94-CA_C0764* vector was digested with SalI, and the operon-containing fragments from each vector were purified on an agarose gel. The 4.9 kb pCLF1 vector[1] was digested with SalI, treated with Antarctic phosphatase and ligated with the previously purified fragment to yield *pCLF-CA_C0764*.

Construction of *pCLF-bcd-etfb-etfa*

The *bcd-etfb-etfa* operon was amplified from the genomic DNA of *C. acetobutylicum MGC∆cac1502* using the *RBS nat bcd_rec_for* and *etfa_rec_rev* primers (Supplementary Table 2). The primers were designed to introduce the native bcd RBS along with the operon and *pCLF-CA_C0764* homologous regions. The purified PCR product was directly ligated with *pCLF-CA_C0764* previously digested with ScaI et SfoI and purified using the GeneArt® Seamless Cloning & Assembly Kit (Thermo Fisher Scientific, Saint Aubin, France). The *pCLF-bcd-etfb-etfa* plasmid was controlled by PCR using the primers *pCLF prom seq_for* and *pCLF term_rev* (Supplementary Table 2), by restriction profiling, and finally by promoter-*bcd-etfb-etfa*-terminator region resequencing.

Construction of the *pCST-LL-CA_C0764*

The *CA_C0764* gene was amplified from the genomic DNA of *C. acetobutylicum ATCC824* using *ccac0764BAMf* and *ccac0764SMAr* primers (Supplementary Table 2). The primers were designed to introduce an RBS region along with the *CA_C0764* gene, as well as placing BamHI and SmaI restriction sites upstream and downstream, respectively. The purified PCR product was further ligated with the product digested with BamHI and SmaI and purified *pthlA-CaHydA-LL-C-Tag* plasmid[2], yielding the *pCST-LL-CAC0764* vector. The *pCST-LL-CAC0764* vector was checked by PCR using pSOSprom_univ and pSOSterm primers (Supplementary Table 2) with the One taq DNA polymerase (New England Biolabs), by restriction profiling, and finally by promoter-*CAC_O764-CST*-terminator region resequencing.

Supplementary Method 2. Transformation procedures

E. coli Top10 (Invitrogen) was transformed using heat shock (30 s at 42°C) following the manufacturer's instructions. MGC*Δcac1502* was electroporated [7], except that unmethylated DNA was used because the *cac1502* gene encoding the type II restriction endonuclease Cac824I was deleted[1]. For transformation with the retargeted *pMTL007::cac-CA_C0764-408s,* after 5 hours of recovery, cells were plated on RCA (Clostridium Nutrient Medium with 15 g/L agar and Fluka (Saint-Quentin Fallavier, France, n°27546) medium supplemented with thiamphenicol (10 µg/mL)). Single colonies were chosen from the plate and streaked separately on an RCA plate with erythromycin (40 µg/mL) to select integrants. The insertion mutants were screened via colony PCR using the *One taq* DNA polymerase (New England Biolabs) and the primers cac0764del_for and cac0764del_rv (Supplementary Table 2) flanking the target site. The PCR products were purified and confirmed by DNA sequencing. One of the colonies was selected to cure the *pMTL007::cac-CA_C0764-408s* plasmid and generate *MGCΔcac1502CA_C0764-*408s::CT. This clone was inoculated into Clostridium Growth Medium (CGM) supplemented with erythromycin (40 µg/mL) for successive subcultures[6]. One hundred microliters of fully grown culture was inoculated into 1 mL of fresh CGM supplemented with erythromycin (40 µg/mL) and grown anaerobically at 37°C for at least 12 hours until full growth was achieved. This transfer process was repeated at least 3 times. The last culture was then plated onto a solid RCA plate supplemented with erythromycin (40 µg/mL). Colonies were restreaked successively onto RCA plates supplemented with thiamphenicol (10 µg/mL) and then onto RCA plates supplemented with erythromycin (40 µg/mL). One erythromycin–resistant and thiamphenicol-sensitive clone was selected and inoculated into 3 mL of SM[8] supplemented with erythromycin (40 µg/mL), grown anaerobically at 37°C for at least 24 hours and transferred to 30 mL of SM supplemented with erythromycin (40 µg/mL). The culture was grown anaerobically at 37°C for

7 days until sporulation, and then the spore suspension was stored at –20°C. Southern hybridization was finally used to validate the presence of a single-intron insertion into the genome of *MGCΔcac1502CA_C0764-408s::CT*.

A similar approach was applied to yield the *MGCΔcac1502-gltB181s::CT* strain. The insertion mutants were screened using the *One taq* DNA polymerase (New England Biolabs) and the primers gltB181s direct and gltB181s reverse (Supplementary Table 2) flanking the target site. The PCR products were purified and confirmed by DNA sequencing.

For transformation with *pCLFCA_C0764* and *pCLF-bcd-etfb-etfa*, after 5 hours of recovery, cells were plated on RCA medium supplemented with thiamphenicol (10 μg/mL). Single colonies were chosen from the plate and streaked separately on an SM plate with thiamphenicol (10 μg/mL).

For transformation with *pCST-LL-CA_C0764* and *pCST-LLbcd-etfb-etfa*, after 5 hours of recovery, cells were plated on RCA medium supplemented with erythromycin (40 μg/mL). Single colonies were chosen from the plate and streaked separately on an SM plate with erythromycin (40 μg/mL).

Supplementary Method 3. Southern blot analysis

Chromosomal DNA (3–6 µg) of both the *MGCΔ1502* and *MGCΔ1502-CA_C0764-408s::CT* strains was digested with the HindIII-HF restriction enzyme and loaded on a 0.8% agarose gel. The transfer was performed in 20xSSC on a nylon membrane. The probe used for hybridization was generated by PCR using ESB universal and ErmB3'- R- F primers (Supplementary Table 2) and then labeled using a DIG High Prime DNA Labeling and Detection Starter Kit II (Roche, Mannheim Germany). The protocols for hybridization and detection were performed according to the Roche instructions.

Supplementary Method 4. RNA isolation, cDNA synthesis and RT-qPCR analysis

After the activation of spores by heat treatment at 80°C for 15 min, both MGC*∆cac1502 and MGC∆cac1502 cac0764-408s::CT* strains were cultivated in duplicate in 60-mL glass vials under strict anaerobic conditions at 37°C in SM[8].

When the OD_{620} of *MGC∆cac1502* and *MGC∆cac1502CA_C0764-408s::CT* cultures reached 2.9 and 1.5, respectively, cultures were sampled, immediately frozen in liquid nitrogen and stored at -80°C until use.

Total RNA isolation was performed as previously described[3]. Briefly, the frozen sampled cultures were ground promptly in a liquid nitrogen-cooled mortar. RNA was extracted using a RNeasy Midi Kit (Qiagen). Contaminant genomic DNA was then removed using the RNase-Free DNase Set (Qiagen) following the manufacturer's instructions, and the total DNase-treated RNA was then purified and concentrated using an RNA Cleanup Kit (Qiagen). RNA quantification was performed using a NanoDrop ND-1000 spectrophotometer (Labtech France) at 260 nm, and purity was analyzed by determining the 260 nm/280 nm ratio (purity > 2.1). RNA integrity was also verified on RNA 6000 Nano Chips using an Agilent 2100 Bioanalyzer (Agilent Technologies). For cDNA synthesis, 1 µg of total RNA was used in a 20 µL reverse transcription (RT) reaction mixture containing iScript Reverse-transcriptase and a blend of oligo(dT) and random hexamer primers using the iScriptTM cDNA Synthesis Kit (Bio-Rad) according to the manufacturer's instructions.

Quantitative real-time PCR (qPCR) was conducted with a MyiQ™ Real Time PCR Detection System (Bio-Rad). Each sample was tested in triplicate in a 96-well plate (Bio-Rad). The reaction

mix (25 µL final volume) consisted of 12.5 µL of SsoAdvanced Universal SYBR Green Supermix (Bio-Rad), 2.5 µL of the primer pair (200 nM final concentration), 2.5 µL of HO and 5 µL of a 1/10 dilution of the cDNA preparation. The absence of possible genomic DNA contamination was checked in each DNase-treated RNA sample. A blank (no template control) was also incorporated in each assay. The thermocycling program consisted of one hold at 95°C for 30 s, followed by 40 cycles of 10 s at 95°C and 30 s at 57°C. After completion of these cycles, melting-curve data were then collected to verify PCR specificity, contamination and the absence of primer dimers.

The fabZ gene (*CA_C3571*) was chosen as an internal control[9]. The primer pairs fabZf/fabZr, gapCf/gapCr and gapNf/gapNr (Supplementary Table 2) were used to amplify the *fabZ*, *gapC* (*CA_C0709*) and *gapN* genes (*CA_C3657*), respectively.

Expression data and associated technical errors were calculated on triplicate experiments using the gene expression module of iQ5 software (Bio-Rad), which uses the model outline on the geNorm website.

Supplementary references

1. Croux, C., Nguyen, N. P. T., Lee, J., Raynaud, C., Saint Prix, F., Gonzales Pajuelo, M., Meynial-Salles, I. & Soucaille, P. Construction of a restriction-less, marker-less mutant useful for functional genomic and metabolic engineering of the biofuel producer *Clostridium acetobutylicum*. *Biotechnol. Biofuels* **9**, 23 (2016).

2. Caserta, G., Papini, C., Adamska-Venkatesh, A., Pecqueur, L., Sommer, C., Reijerse, E., Lubitz, W., Gauquelin, C., Meynial Salles, I., Pramanik, D., Artero, V., Atta, M., del Barrio, M., Faivre, B., Fourmond, V., Léger, C. & Fontecave, M. Engineering an [FeFe]-Hydrogenase: Do accessory clusters influence O_2 resistance and catalytic bias ? *J. Am. Chem. Soc.,* **140**, 5516-5526 (2018).

3. Yoo, M., Bestel-Corre, G., Croux, C., Riviere, A., Meynial-Salles, I. & Soucaille, P. A quantitative system-scale characterization of the metabolism of *Clostridium acetobutylicum*. *mBio* **6**, e01808–15 (2015).

4. Heap, J. T., Pennington, O. J., Cartman, S. T., Carter, G. P. & Minton, N. P. The ClosTron: a universal gene knock-out system for the genus *Clostridium*. *J. Microbiol. Methods*. **70**, 452-64 (2007).

5. Perutka, J., Wang, W., Goerlitz, D. & Lambowitz, A. M. Use of computer-designed group II introns to disrupt *Escherichia coli* DExH/D-box protein and DNA helicase genes*. J. Mol. Biol.* **336**, 421-39 (2004).

6. Dusséaux, S., Croux, C., Soucaille, P. & Meynial-Salles, I. Metabolic engineering of *Clostridium acetobutylicum* ATCC 824 for the high-yield production of a biofuel composed of an isopropanol/butanol/ethanol mixture. *Metab. Eng*., **18**,1-8 (2013).

7. Mermelstein, L. D., Welker, N. E., Bennett, G. G. & Papoutsakis, E.,T**.** Expression of cloned homologous fermentative genes in *Clostridium acetobutylicum* ATCC 824. *Biotechnology* **10**, 190-195 (1992).

8. Girbal, L. & Soucaille, P. Regulation of *Clostridium acetobutylicum* metabolism as revealed by mixed-substrate steady-state continuous cultures: Role of NADH/NAD ratio and ATP pool. *J. Bacteriol.* **176,** 6433–6438 (1994).

9. Jones, S. W., Paredes, C. J., Tracy, B., Cheng, N., Sillers, R., Senger, R. S. & Papoutsakis, E. T. The transcriptional program underlying the physiology of clostridial sporulation. *Genome Biol.* **9**, R114 (2008).
